# Meteorological Drivers of Extremes in Daily Stem Radius Variations of Beech, Oak, and Pine in Northeastern Germany: An Event Coincidence Analysis

**DOI:** 10.3389/fpls.2016.00733

**Published:** 2016-06-03

**Authors:** Jonatan F. Siegmund, Tanja G. M. Sanders, Ingo Heinrich, Ernst van der Maaten, Sonia Simard, Gerhard Helle, Reik V. Donner

**Affiliations:** ^1^Research Domain IV—Transdisciplinary Concepts and Methods, Potsdam Institute for Climate Impact ResearchPotsdam, Germany; ^2^Institute of Earth and Environmental Science, University of PotsdamPotsdam, Germany; ^3^Thünen Institute of Forest EcosystemsEberswalde, Germany; ^4^Department 5 Geoarchives, Helmholtz Centre Potsdam, GFZ German Research Centre for GeosciencesPotsdam, Germany; ^5^Institute of Botany and Landscape Ecology, University of GreifswaldGreifswald, Germany

**Keywords:** dendrometer measurements, event coincidence analysis, climate extremes, growth response

## Abstract

Observed recent and expected future increases in frequency and intensity of climatic extremes in central Europe may pose critical challenges for domestic tree species. Continuous dendrometer recordings provide a valuable source of information on tree stem radius variations, offering the possibility to study a tree's response to environmental influences at a high temporal resolution. In this study, we analyze stem radius variations (SRV) of three domestic tree species (beech, oak, and pine) from 2012 to 2014. We use the novel statistical approach of event coincidence analysis (ECA) to investigate the simultaneous occurrence of extreme daily weather conditions and extreme SRVs, where extremes are defined with respect to the common values at a given phase of the annual growth period. Besides defining extreme events based on individual meteorological variables, we additionally introduce conditional and joint ECA as new multivariate extensions of the original methodology and apply them for testing 105 different combinations of variables regarding their impact on SRV extremes. Our results reveal a strong susceptibility of all three species to the extremes of several meteorological variables. Yet, the inter-species differences regarding their response to the meteorological extremes are comparatively low. The obtained results provide a thorough extension of previous correlation-based studies by emphasizing on the timings of climatic extremes only. We suggest that the employed methodological approach should be further promoted in forest research regarding the investigation of tree responses to changing environmental conditions.

## 1. Introduction

During the past 15 years the systematic installation and operation of dendrometers and analysis of the obtained data has received increasing interest in forestry sciences. While the first attempts (Friedrichs, 1897) to use dendrometer data to analyze tree response to environmental conditions were clearly limited by the technical conditions of early instruments, recent developments in the production of modern automated high-precision dendrometers offer the ability to generate dendrometer time series at very high temporal and spatial resolution (Drew and Downes, 2009). The detailed representations of activity in the tree stem—shrinkage, recovery, and swelling cycles—allow for a high-temporal investigation of the tree stem as well as long-term morphological and short-term physiological dynamics (Zweifel and Häsler, 2001; Deslauriers et al., 2003; McLaughlin et al., 2003; Bouriaud et al., 2005; Daudet et al., 2005; Vieira et al., 2013). Where additional environmental information is available, dendrometer data can provide information on the tree stems response to external factors, especially meteorological conditions (McLaughlin et al., 2003; Denneler et al., 2010; Miralles-Crespo et al., 2010; Oberhuber and Gruber, 2010; Jezik et al., 2011; Butt et al., 2014). Investigations such as these are also important in order to better understand the diurnal cycle of sap flow and leaf water potential (Drew and Downes, 2009).

Beyond the fundamental understanding of tree functioning, dendrometer data can also indirectly provide important information on the carbon cycle at the local, regional or global level. Even though stem radius does not allow estimates of total cell numbers, it is an important proxy for a forest's above ground biomass (Schulte-Bisping et al., 2012), because it can help to determine the wood volume available for the fixation of carbon (Cuny et al., 2015).

To the authors' best knowledge, almost all above-mentioned studies have investigated dendrometer data using classical statistical tools like linear correlation analysis or linear multiple regression. These powerful methodological approaches have led to an understanding of the relationship between stem size changes and various environmental parameters. Yet, correlation-based approaches generally take all parts of the distributions of two variables of interest into account and therefore describe the joint behavior of these variables. A crucial question only sporadically addressed so far is how tree stem radius variations (SRVs) are linked to extreme weather conditions. This question gains special importance, since recent climate projections suggest a rising frequency and severity of meteorological extreme events for many parts of the world (Barriopedro et al., 2011; IPCC, 2013; Petoukhov et al., 2013). Consequently, analyzing the impact of such extremes on tree SRVs, on the one hand can help to better understand an event's impact on tree functionality and carbon cycle, and on the other hand, because different tree species may be impacted differently, to generate recommendations for future forest management (Spathelf et al., 2014). New findings concerning the response of stem radius to extreme meteorological conditions would also help to improve existing climate-growth-models like TREERING (Fritts et al., 1999) or CAMBIUM (Drew, 2007).

An important study addressing the response of stem-size fluctuations and tree radius growth to climatic extremes using a large number of dendrometer data sets was recently published by Butt et al. (2014). However, this study did not report explicit results regarding the response of the tree stem to extreme meteorological conditions, due to the fact that they have only used ordinary linear regression to analyze the data.

In this study, we employ the novel methodological approach of event coincidence analysis (ECA) to quantify possible simultaneities between extraordinary daily stem variations and extraordinary meteorological conditions. Here, the commonly used term *extreme* is replaced by *extraordinary*, referring to the upper and lower tails of the empirical distributions of the variables of interest. Due to the comparatively short investigation period of 3 years (and only 8 years of climatological data), “real” extreme events are difficult to identify or define and therefore the investigation of extraordinary conditions shall represent a tree's reaction to the tails of the empirical distribution of weather events, which may well be exceeded by future developments under climate change. Therefore, conclusions from this study's results on trees' reaction on weather extremes should be made unter consideration of the used definition of “extraordinary events.”

Taking into account the existing literature on SRVs and their relation to meteorological conditions we expect that extraordinary climatic events, specifically temperature events, and extraordinary dendrometer variations should occur simultaneously. Additionally, it can be expected that there are clear inter-species differences concerning the reaction to extraordinary meteorological events.

## 2. Materials and methods

### 2.1. Study area and data sampling

#### 2.1.1. Study area

The study site was close to Lake Hinnensee (53.33°N, 13.19°E) in the northeastern part of Germany. The site is located within the Müritz National Park. Large parts of this protected area have been classified as UNESCO World Natural Heritage in 2011. The park is characterized by 200–300 years old mixed beech, pine and oak stands. The climate is semi-continental, typical for central Europe, with a mean annual temperature of about 8°C and an annual precipitation between 550 and 650 mm. The soil at the study site as well as at the meteorological station (see Section 2.2.2) is a brunic arenosol on sand of outwash plains, characterized by strong hydraulic permeability Müller (2014).

#### 2.1.2. Data sampling

The dendrometer data were collected for three tree species: European beech (*Fagus sylvatica*), Scots pine (*Pinus sylvestris*), and Sessile oak (*Quercus petraea*). The distances between the individual trees range from ca. 4 to 20 m and the selection of trees was based on the canopy status of the individual trees (i.e., only dominant trees or co-dominant trees were equipped with dendrometers). This study focuses on the species' response to meteorological conditions, hence the dendrometer data are not differentiated according to the relative positions of the individual trees in landscape (see Section 2.3), but trees were selected along a transect from the lake shore to a terrace ~15 m above Lake Hinnensee, in order to sample a possibly large variation of individual local stand variations. The equipped trees have an average height of 26 m with diameters between 50 and 70 cm at breast height.

For each tree species, 10 individuals were equipped with Ecomatik DR point radius dendrometers (Ecomatik GmbH, 2015) installed at 1.2 m height. The sensors were installed at the north face of the trunks in order to avoid direct irradiation. They have a temperature coefficient < 0.1m/K. Bark was mostly removed from pine and oak trees prior to setup. SRVs were measured at a temporal resolution of 30 min over a 3-years period between 2012 and 2014.

### 2.2. Data preprocessing

#### 2.2.1. Dendrometer data

After a comprehensive quality check, the raw dendrometer data were pre-processed using the following three steps:

1. In a first step, the 30-min resolution dendrometer data were used to calculate daily SRVs. Rather then using a stem cycle approach (Deslauriers et al., 2003; Köecher et al., 2012; Vieira et al., 2013) which distinguishes between phases of contraction, expansion, and stem radius increment, we calculated day-to-day SRVs as the first-order differences between mean daily dendrometer recordings. Similar methods were used, for example, by Bouriaud et al. (2005) or van der Maaten et al. (2013). This procedure results in positive and negative values following a clear seasonal cycle. In contrast to van der Maaten et al. (2013), the daily residuals were not normalized by the season's total growth due to the age maturity of the investigated trees.
2. As a second step, a two-sided sliding window mean (window size of 15 days) was subtracted from the resulting daily SRVs in order to account for the seasonal cycle. The resulting residuals represent the daily stem increments of a tree as deviations the from the 15-day mean. Finally, the investigation period was defined from April 1st to September 30th of each year to cover the entire growth period for each species. A sliding window approach (see Section 2.3) was applied to produce comparable results which are not shifted against each other by species or year.
3. In order to transform the dendrometer time series into event time series, we applied a 90th and 10th percentile threshold to the daily increments. Values exceeding the 90th percentile of all days of the investigation period were defined as extraordinary positive SRV events, whereas days lower than the 10th percentile were defined as negative events. This event definition results in 55 positive and 55 negative events during the 3-years period for each individual tree. Due to the usage of residuals with respect to the “normal” seasonal behavior, these events are approximately homogeneously distributed within each year (not shown) and the specific timings of the individual events are determined by environmental conditions. An important exception is a very dry period during the summer of 2013 during which only a few strong positive precipitation events were observed.

#### 2.2.2. Meteorological data

In order to define days with extraordinary meteorological conditions, data from a nearby meteorological station in Serrahn (at a distance of less than 2 km from the study site) were used. The soil conditions at the dendrometer site and the meteorological station site are comparable, but not identical. Systematically differing soil temperatures, for example, can not be excluded. In addition to air temperature and precipitation, the station provides information on (relative air) humidity, soil temperature, air pressure and incoming solar radiation. The data set is available starting January 2006 at a temporal resolution of 1 h. Similar to the daily SRVs, the meteorological data were pre-processed in order to identify events of extraordinary daily meteorological conditions:

1. The hourly information was aggregated to daily minimum, mean, and maximum values. Observations of air pressure and minimum radiation were not used since no meaningful results are expected by using these variables.
2. The daily meteorological information was transformed to *z-scores*. Since the time series length of the meteorological station spans only 8 years, the z-scores calculation differs slightly from the classical approach: for each day of the year the mean over all 8 years was additionally averaged over sliding windows (with a window length of 45 days for precipitation, 15 days for all other variables). Then, the resulting mean seasonal cycle as well as the estimated seasonal cycle in variance were used for obtaining z-scores.
3. Finally, the resulting z-scores were transformed to event time series by applying a 90th and 10th percentile threshold as in the case of the dendrometer data. Due to the application of these percentiles for threshold definition, the number of events in each meteorological variable is also 55 (each negative and positive).

The following meteorological variables were used: air temperature at 2 m (*T*_*min*_, *T*_*mean*_, and *T*_*max*_), land surface air temperature at 5 cm (*LST*_*min*_, *LST*_*mean*_, and *LST*_*max*_), soil temperature in 20 cm depth (*ST*_*min*_, *ST*_*mean*_, *ST*_*max*_), relative humidity (*rH*_*min*_, *rH*_*mean*_, *rH*_*max*_), total precipitation (*P*_*tot*_) and incoming solar radiation (*RAD*_*mean*_ and *RAD*_*max*_). Many of these variables are highly correlated among each other. Hence, for a study using classical statistical approaches a principal component analysis (as performed by, e.g., Beck et al., 2013) would be appropriate in order to reduce the number of meteorological observables (i.e., to eliminate collinear variables). However, in this study the novel statistical approach of ECA (see Section 2.3) is applied, where the reduction of dimensions based on their common mean behavior (correlation) would not be useful, since the information of interest (timing of extraordinary events) could eventually get lost. Additionally, this study is particularly focused on the different variations of air, surface and soil temperatures, for example. As an alternative, a dimensionality reduction based on event coincidence rates (see Section 2.3) replacing classical linear correlations as similarity measure could be performed as a preparatory step. The utilization of such a novel approach is, however, beyond the scope of the present study.

### 2.3. Statistical analysis

#### 2.3.1. Bivariate event coincidence analysis

In order to investigate the simultaneity of events in meteorological variables and SRV, we apply *event coincidence analysis (ECA)* (Donges et al., 2016), a novel yet conceptually simple statistical concept. In its basic setting, ECA considers two sequences of events of different types (A and B). As the hypothesis to be tested, events of type B are considered to causally influence the timing of events of type A. To cope with realistic scenarios, ECA allows to not only trivially quantify the number of exactly simultaneous occurrences of events of both types, but to consider also lagged as well as time-uncertain responses. For the latter purpose, a time lag parameter τ as well a temporal tolerance window Δ*T* can be additionally taken into account. Then, ECA counts how often events of types A and B occur with a mutual delay τ in both sequences within a certain temporal tolerance Δ*T*. The resulting number of “event coincidences,” divided by the total number of events in one of the series is called the *coincidence rate r*.

**Figure 1:**
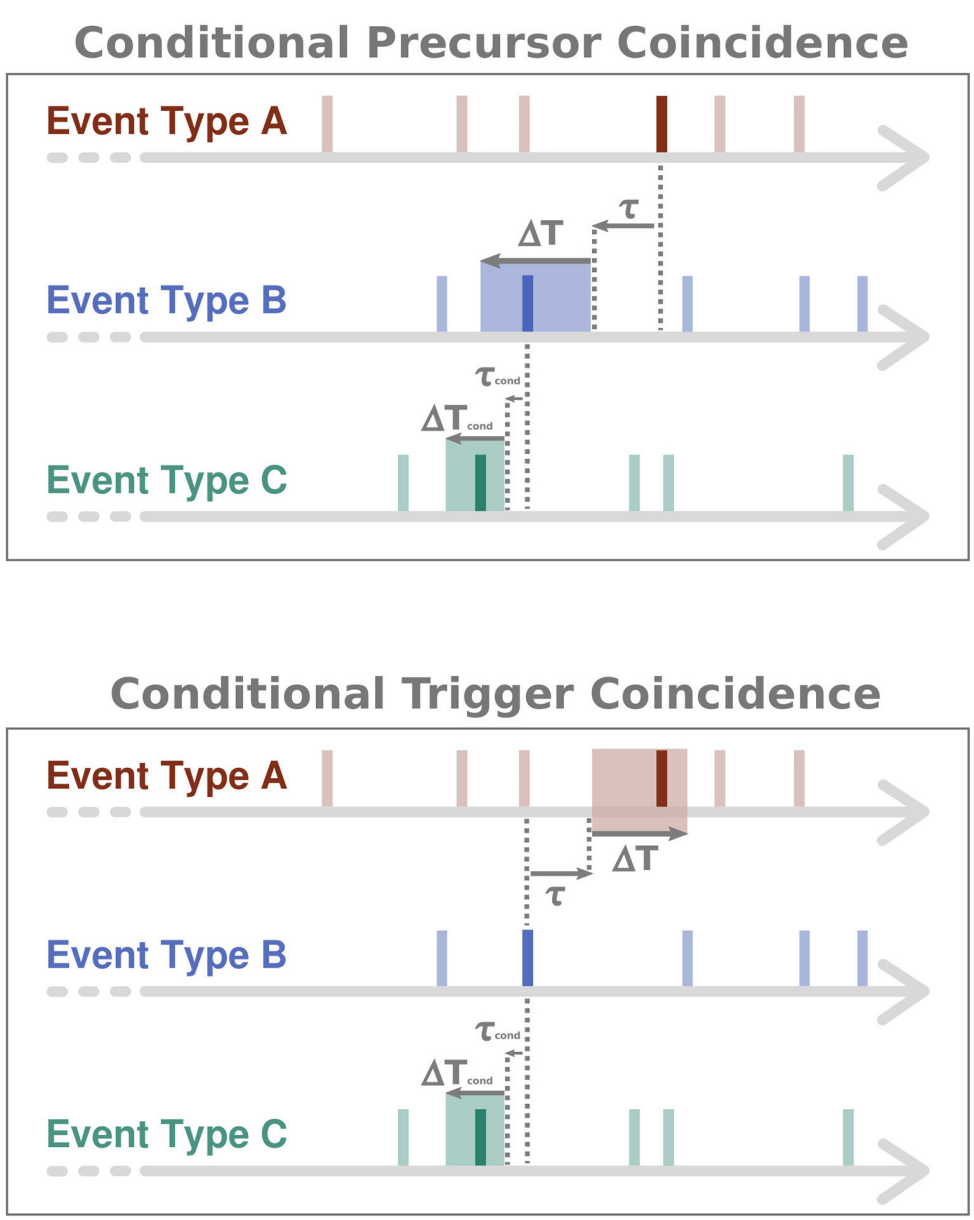
**Schematic illustration of (conditional) ECA**. In the conditional case, only those events of type B are considered as coinciding with events of type A, that are preceded by at least one event of type C. This conditioning is expressed by a precursor coincidence between events of type B and type C. While Δ*T* and τ denote the tolerance window and time lag parameter for counting coincidences between events of types A and B, Δ*T*_*cond*_ and τ_*cond*_ are the respective parameters for the conditioning of events of type B on events of type C.

Since the statistical analysis described above is not symmetric, ECA defines two distinct types of coincidence rates, *r*_*p*_ (*precursor coincidence rate*) and *r*_*t*_ (*trigger coincidence rate*). Here, *r*_*p*_ is defined as the number of event coincidences divided by the number *N*_*A*_ of events of type A, describing the fraction of events of type A that have been preceded by at least one event of type B. In turn, *r*_*t*_ is defined as the number of event coincidences divided by the number *N*_*B*_ of events of type B, thereby describing the fraction of events of type B that have been followed by (and, thus, potentially triggered) at least one event of type A. When using τ ≠ 0, this differentiation is essential. A schematic illustration of the two different types of coincidence rates can be found in Figure 1.

In addition to the simple calculation of coincidence rates, the R package CoinCalc used in this work for performing the corresponding analyses provides different possibilities to test whether the empirically found coincidence rates are significantly different from what could result from two independent random event sequences (Siegmund et al., 2016). In this work, we will exclusively utilize an analytical significance test based on the assumption of Poissonian event statistics (Donges et al., 2011, 2016; Siegmund et al., 2015).

#### 2.3.2. Conditional and joint event coincidence analysis

As a thorough extension of the basic ECA method for two event sequences, in this work, we introduce new multivariate generalizations of ECA termed *conditional event coincidence analysis (CECA)* and *joint event coincidence analysis (JECA)*. While the above formulation of bivariate ECA is sufficient for many applications (e.g., in Donges et al., 2011), in order to analyze the reaction of ecological variables to extraordinary meteorological conditions, it may be important to additionally consider the conditioning of events on specific situations governed by a third observable, i.e., the case that events of type B appear simultaneously with events in series A if and only if also, one or more events of a third type C take place. This conditioning could, for instance, be relevant to account for the interplay between temperature and moisture or between moisture and radiation.

The conditioning of events of type B by events of type C can be described by the precursor coincidence between B and C. Therefore, the *conditional precursor coincidence rate r*_*cp*_ and the *conditional trigger coincidence rate r*_*ct*_ between A and B can be defined (in analogy to *r*_*p*_ and *r*_*t*_ as mathematically defined by Equations (3) and (4) in Donges et al., 2016) as:

(1) $$\begin{array}{l} r_{cp}(\Delta T,\tau ,\Delta T_{cond},\tau_{cond})=\\ \;\frac{1}{N_{A}}\sum_{i\;=\;1}^{N_{A}}\Theta [\sum_{j\;=\;1}^{N_{B}}\Theta \left[\sum_{k\;=\;1}^{N_{C}}{𝟙}_{\left[0,\Delta T_{cond}\right]}((t_{j}^{B}-\tau_{cond})-t_{k}^{C})\right]\\ \;\;{𝟙}_{\left[0,\Delta T\right]}((t_{i}^{A}-\tau )-t_{j}^{B})] \end{array}$$

and

(2) $$\begin{array}{l} r_{ct}(\Delta T,\tau ,\Delta T_{cond},\tau_{cond})=\\ \;\frac{1}{N_{B,cond}}\sum_{j\;=\;1}^{N_{B}}\Theta [\sum_{i\;=\;1}^{N_{A}}\Theta \left[\sum_{k\;=\;1}^{N_{C}}{𝟙}_{\left[0,\Delta T_{cond}\right]}((t_{j}^{B}-\tau_{cond})-t_{k}^{C})\right]\\ \;{𝟙}_{\left[0,\Delta T\right]}((t_{i}^{A}-\tau )-t_{j}^{B})], \end{array}$$

respectively. Here, $\left\{t_{i}^{A}\right\}$, $\left\{t_{j}^{B}\right\}$ and $\left\{t_{k}^{C}\right\}$ are the timings of the events of types A, B and C, respectively, *N*_*C*_ is the number of events of type C, Δ*T*_*cond*_ is an additional tolerance window for the condition, τ_*cond*_ a time lag parameter for the condition, and *N*_*B, cond*_ is the number of conditional events of type B, i.e., the number of events of type B that show a precursor coincidence with at least one event of type C. Θ(·) denotes the Heaviside function (i.e., takes a value of one whenever the argument is non-negative, and zero otherwise) and 1_*I*_ the indicator function of the interval *I* (i.e., takes a value of one whenever the argument is within *I*, and zero otherwise), respectively. In order to visualize the basic idea of the corresponding CECA, Figure 1 illustrates the approach in a conceptional way.

Using the definitions in Equations (1) and (3), the conditional precursor coincidence rate describes the fraction of events in series A, that appear simultaneously with C-conditioned events of type B, and the conditional trigger coincidence rate is the fraction of C-conditioned events of type B that are followed by at least one event in series A. In the special case of a simultaneous occurrence of events of types B and C (i.e., τ_*cond*_ = 0), we obtain a setting referred to as JECA.

#### 2.3.3. Methodological setting in the present study

For the application of ECA and CECA/JECA, we dissect the 1095 days period from 2012 to 2014 by sliding windows. For the (bivariate) ECA, the window length is chosen as 61 days with a step size of 5 days, resulting in 75 windows per growing season (1 April to 30 September), where each window contains six events on average. The window length of 61 days is a compromise between a desired high temporal resolution and a possible large window size necessary to produce robust statistics. The step size of 5 days was selected in order to mimimize the computational demand. For the (multivariate) CECA/JECA, the window length is extended to 91 days including nine events on average, since due to the additional conditioning, the number of events in the meteorological variables decreases markedly. In order to cope with the high computational demand of CECA/JECA for 15 variables, the window step size was increased to 10 days, resulting in 15 windows per season.

As a first step, using the sliding window approach, ECA was performed between each of the 15 individual meteorological variables and each tree's SRV series across each window separately. For every window, the fraction of trees with a significant number of coincidences was taken as a proxy describing the reaction of the species to the considered meteorological events. Subsequently, JECA was performed between the dendrometer data and all pairs of meteorological variables. As a consequence, the chosen analysis setting results in 15 × 14/2 = 105 different variable combinations. Note that for τ_*cond*_ ≠ 0, i.e., mutually shifted occurrences of events in the two considered meteorological variables, the number of combinations to be considered in an actual CECA would be twice as large. Therefore, we do not consider mutually conditioned events in this pilot study, but leave a corresponding detailed investigation as a subject of future work. Besides this, we do not consider the possible extension of multivariate conditions (involving different meteorological variables), which would be straightforward yet lead to an even larger combinatorial variety of different cases to be studied.

In all analyses discussed in the remainder of this work only the (conditional) precursor coincidence rates are considered unless stated otherwise.

#### 2.3.4. Cluster analysis

In order to analyze the simultaneity of event timings between the individual trees, we additionally use the well established approach of hierarchical cluster analysis with complete linkage. Core of the concept of cluster analysis (in this case for dendrometer time series) is a similarity measure, calculated between all possible combinations of individual time series. This similarity measure is, classically, a correlation coefficient. In this study, we additionally introduce the application of event coincidence rate as similarity measure for the cluster analysis. The calculation of the event coincidence rate between each pair of dendrometer event time series follows along the above mentioned approach, using τ = Δ*T* = 0. Due to the above described data preprocessing, there is no difference between precursor and trigger event coincidence rate.

## 3. Results

### 3.1. Event coincidence analysis with individual meteorological variables

**Figure 2:**
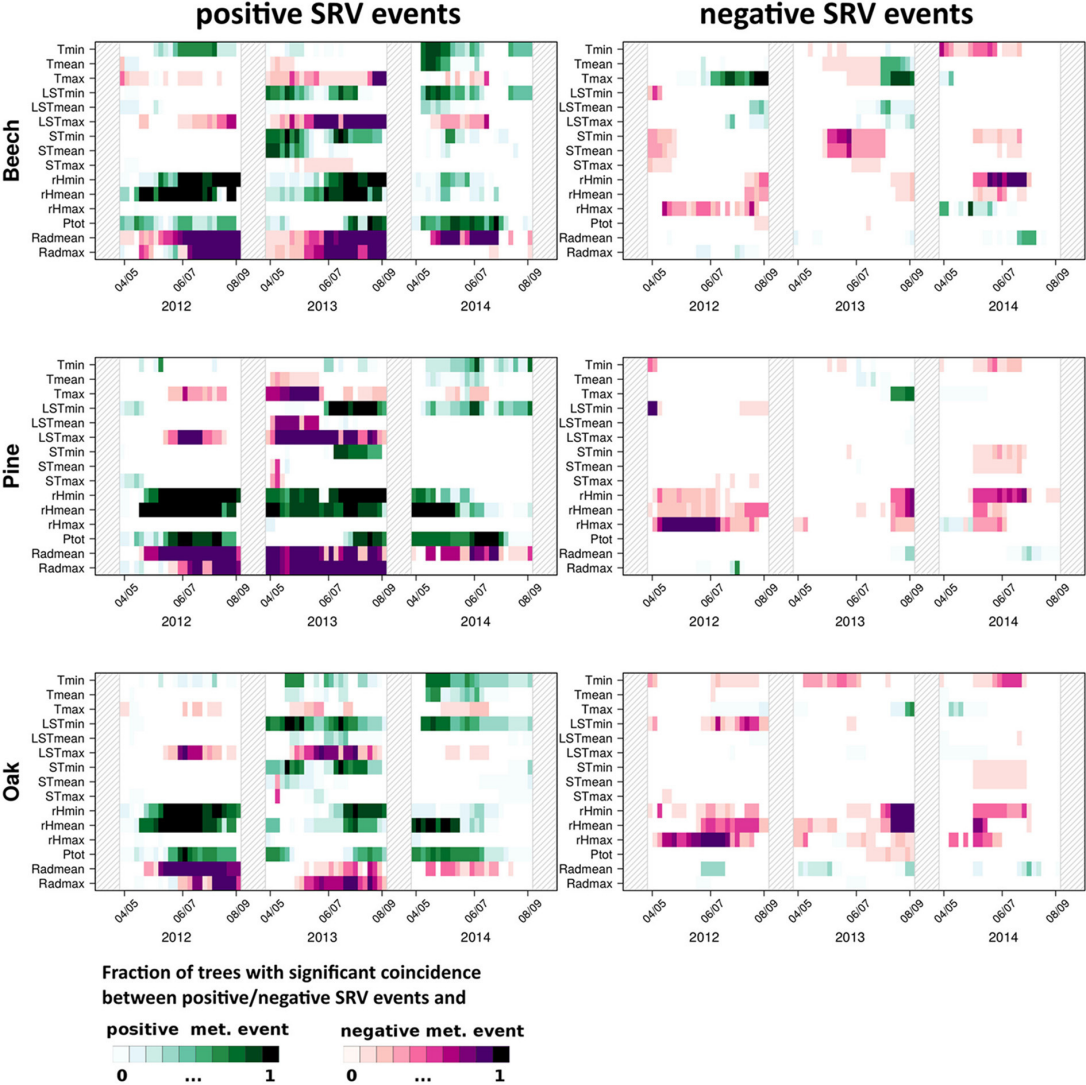
**Results of precursor ECA (Δ*T* = 0 and τ = 0) between positive (left panels) and negative SRV events (right panels) and extraordinarily high (above the 90th percentile, “positive meteorological events”) and low (below the 10th percentile, “negative meteorological events”) expressions of 15 meteorological variables for beech, oak, and pine during the study period from 2012 to 2014**. Colors indicate the fraction of significant coincidences (at α = 0.05 confidence level). The dates at the x-axes denote the centers of the sliding windows (61 days).

Figure 2 shows the fraction of trees with significant precursor coincidence rates between extraordinary positive/negative SRVs and positive/negative events in each of the 15 meteorological variables at the same day (Δ*T* = τ = 0).

For positive SRV events (Figure 2, left panel), five main observations are made: (i) *T*_*min*_ and *T*_*max*_ events have an opposite effect in almost all years and for all tree species. Extraordinary positive SRV events mainly coincide with *T*_*min*_ values above the 90th percentile and *T*_*max*_ values below the 10th percentile. The same observation is also clearly visible for the land surface temperature. (ii) Extraordinary soil temperature generally has a much lower impact on positive SRV events than extraordinary air temperature. Except for 2013 for beech, *ST*_*mean*_ and *ST*_*max*_ only rarely show coincidences with positive or negative SRV events, while at least extraordinary positive minimum soil temperatures often coincide with positive SRV events. (iii) Extraordinary high values of relative humidity coincide with positive SRV events in all years and for all species. The fraction of trees showing significant coincidences has been slightly reduced in 2014 in comparison with the other 2 years. (iv) Extraordinary high precipitation values do almost continuously coincide with positive SRV events. An important exception is the summer of 2013, where neither tree species showed a corresponding significant relationship. (v) Extraordinary low values of mean and maximum radiation, generally show pronounced coincidences with SRVs above the 90th percentile.

In comparison to positive SRV events, negative events clearly show fewer significant coincidences with extraordinary meteorological conditions (Figure 2, right panel). However, two features can be highlighted: (i) For beech, values above the 90th percentile of the maximum temperature strongly coincide with strong negative dendrometer anomalies in 2012, less distinct in 2013, and hardly ever in 2014. In turn, negative oak and pine SRV events do not coincide significantly with maximum temperature events. (ii) Extraordinary low values of relative humidity very often coincide with negative SRV events for all species. This feature is variably expressed during the 3 years of observations but particularly evident in 2012. In contrast, during 2014 maximum relative humidity events (very wet days) significantly coincide with negative beech SRV events.

**Figure 3:**
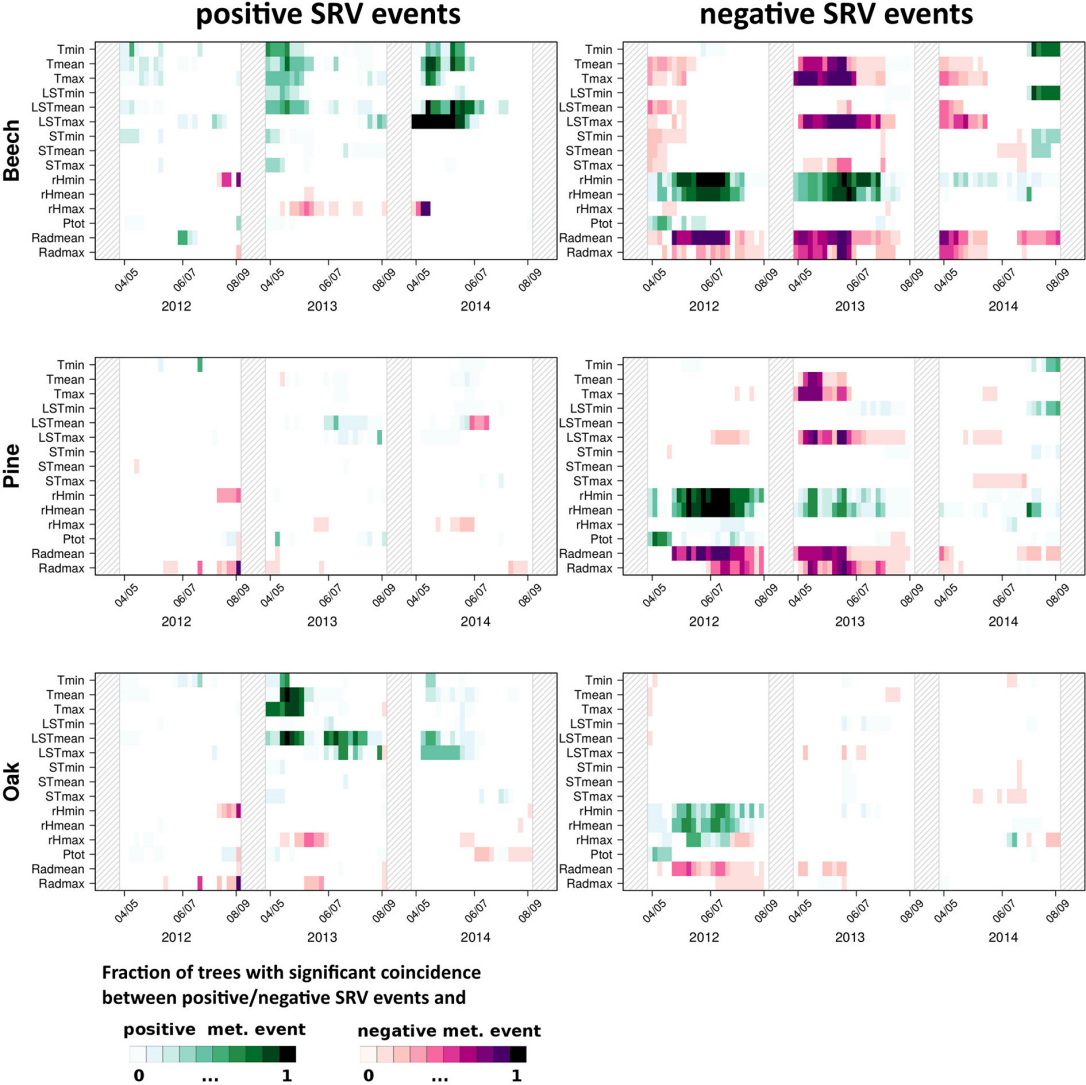
**As in Figure 2 but with Δ*T* = 1 day and τ = 1 day**.

In addition to the consideration of exactly simultaneous coincidences as described above, Figure 3 shows the results of precursor ECA using a tolerance window spanning the previous 2 days (Δ*T* = τ = 1 day), i.e., this kind of analysis takes into account responses with a time lag of 1 and 2 days. While in this case, positive dendrometer anomalies only show few coincidences with extraordinary meteorological conditions, on the contrary extraordinary negative stem size changes exhibit three major patterns: (i) Maximum land surface and maximum air temperature events show coincidences of their highest values with negative SRV events, which is especially distinct for beech in 2013. (ii) Extraordinary minimum and mean relative humidity values above the 90th percentile show clear coincidences with negative SRV events in 2012 and 2013 for all tree species. (iii) Extraordinary low mean and maximum radiation values coincide with negative SRV events as well. The two last features are mainly visible for beech and pine and are most distinct in 2012 and 2013. Notably, these results are similar to the previous analysis for positive SRV events when using Δ*T* = τ = 0 (see Figure 2).

### 3.2. Joint event coincidence analysis for paired meteorological variables

Due to the large number of possible combinations between meteorological variables, the figures showing the results of JECA are provided in the supplementary material. The following analysis concerns *equally directed events* if the values of both meteorological variables under study either both exceed their respective upper threshold value or both fall below their respective lower threshold value used for the definition of events. In the other case where one variable takes extraordinarily high values and the other extraordinarily low ones or vice versa, we will speak of *oppositely directed events*.

#### 3.2.1. Beech

Figure S1 shows the results of JECA between beech SRV events and equally as well as oppositely directed events of each pair of meteorological variables, respectively. Six main observations are to be highlighted (which are also evident for the two other tree species but to different degrees): (i) In 2012, extraordinary high minimum temperatures in combination with extraordinary high relative humidity strongly coincide with positive SRV events. (ii) Various combinations of all temperature variables coincide with positive SRV events, but for beech almost only in 2014. (iii) Extraordinary low maximum land surface as well as low maximum air temperature events in combination with extraordinary low mean and maximum radiation coincide with positive SRV events, mainly in 2012 and 2013. (iv) Extreme precipitation plays a rather minor role for beech SRV events when applying JECA for equally directed meteorological extremes. In turn, the combinations of extraordinary low maximum air or land surface temperature with extraordinary high precipitation or extraordinary high air humidity strongly coincide with positive beech SRV events. In addition, (v) extraordinary high minimum temperature values together with extraordinary low radiation, and (vi) extraordinary humid conditions (in terms of strong precipitation or high air humidity) again together with extraordinary low radiation also coincide with positive beech SRV events.

The investigation of negative beech SRV events using JECA shows hardly any significant coincidences (see Figure S2). Whenever evident, the behavior is simply opposite to the effects of positive change anomalies and shall therefore not be further detailed here.

#### 3.2.2. Oak

The results of JECA for oak SRV events are provided in Figure S3. The left panel shows again that (i) during a period in early summer 2012 extraordinary high minimum temperatures in combination with extraordinary high air humidity coincide with positive SRV events, and (ii) various combinations of extraordinary high temperatures coincide with positive oak SRV events as well. Unlike beech, oak stem variations also show this feature in 2013. (iii) Additionally, extraordinary low maximum air and land surface temperatures together with extraordinary low radiation coincide with positive SRV events. (iv) Extraordinary high minimum and mean temperatures together with extraordinary precipitation events appear simultaneously with positive SRV events mainly in 2014 and partly in 2013. In contrast to this, similar to beech SRV events, negative maximum temperatures events with co-occuring extraordinary humid conditions coincide with positive oak SRV events in all 3 years. The features (v) and (vi) are very similar to what has been illustrated in Figure S1 for beech.

For joint coincidences between negative oak SRV events and pairs of meteorological variables (Figure S4), the results are hardly significant in general. However, three specific observations can be made regarding conditions in late summer 2013: (i) Negative SRV events coincide with very dry conditions - indicated by the various combinations of extraordinary low humidity as well as (ii) positive maximum temperature events together with negative humidity events and (iii) negative humidity with positive mean and/or maximum radiation events.

#### 3.2.3. Pine

The features (i), (ii), and (iii) indicated in Figure S5 are very similar to the corresponding features in Figure S1. Yet, feature (i) for positive pine SRV events is additionally visible in 2013. The positive impact of very humid conditions (iv) in terms of combinations of extraordinary high air humidity and extraordinary strong precipitation as well as of low maximum air and land surface temperatures and strong precipitation or high air humidity is clearly visible in all 3 years of observations. The features (v) and (vi) reported above are also clearly visible and in general more distinct than for beech and oak.

Negative pine SRV events (Figure S6) coincide with the same extraordinary meteorological conditions that were observed for oak.

### 3.3. Positive SRVs and time lagged negative SRVs

As already mentioned in Section 3.1, when comparing the results of ECA without time lag and tolerance window with the results using Δ*T* = τ = 1 day, one very important feature is that relative humidity and radiation show coincidences for both, positive (Δ*T* = τ = 0) and negative (Δ*T* = τ = 1 day) SRV events. A very similar finding was also reported by van der Maaten et al. (2013), based on correlation analysis. The interpretation of this observation leads to the hypothesis that in many cases, after a positive SRV event a negative event occurs during one of the two following days. In order to further test this hypothesis, we also performed ECA between negative SRV events and positive SRV events for previous days. For this purpose, we used negative events (see Section 2) as event series A and positive events as series B with Δ*T* = 2 days and τ = 1 day. In this analysis a precursor coincidence is found, if a negative event is preceded by a positive event at one of the three previous days, whereas a trigger coincidence is observed if a positive event is followed by a negative event during one of the following 3 days. This analysis was performed for all tree species and for each year separately. Table 1 summarizes the results which indicate very high rates of both trigger and precursor coincidence. Notably, a very high fraction of positive SRV events (up to 64%) precede negative events at one of the three consecutive days. In all these cases, the observed positive SRV events very likely do not correspond to irreversible growth, but rather reversible swelling. On the other hand, the remaining positive events (40–50%) have not been followed by negative events, i.e., either not followed by stem radius decrease at all or by a gradual decrease that is not identified as extraordinary with the employed event definition.

**Table 1:** **Mean precursor and trigger coincidence rates (10 trees per species) between negative (event type A) and positive SRV events (event type B), using Δ*T* = 2 days and τ = 1 day (i.e., a negative event following a positive events)**.

	**Trigger**			**Precursor**		
	**2012**	**2013**	**2014**	**2012**	**2013**	**2014**
Beech	0.53	0.39	0.44	0.49	0.38	0.55
Pine	0.64	0.54	0.45	0.57	0.58	0.50
Oak	0.60	0.45	0.31	0.51	0.42	0.34

To further investigate this question, we additionally used JECA with the same setup, where the positive SRV events (series B) have been observed in parallel with extraordinarily high rH values (series C). Table 2 summarizes the results of this analysis. Notably, the observed joint trigger coincidence rates are clearly higher than the trigger coincidence rates in Table 1 which implies that positive SRV events induced by high air humidity are more likely to be followed by negative SRV events than other positive SRV events. A possible scenario consistent with this finding would be thunderstorms during relatively dry and/or hot periods, where wet conditions induce positive SRVs and rapid hydrological processes return to dry soil conditions very quickly again. Moreover, we find that the joint trigger coincidence rates are distinctively higher than the joint precursor coincidence rates. This indicates that most of the humidity-induced positive SRV events have triggered negative events, but only a smaller fraction of negative events have been preceded by humidity-induced positive SRV events. This finding suggests, that there are different types of positive SRV events (followed or not followed by negative SRV events events) and different types of negative SRV events events (preceded or not preceded by positive SRV events).

**Table 2:** **Mean joint precursor and joint trigger coincidence rates (10 trees per species) between negative SRV events (series A), positive SRV events (series B) and extraordinarily high *rH*_*mean*_ events (series C), using Δ*T* = 2 days, τ = 1 day, Δ*T*_*cond*_ = 0 and τ_*cond*_ = 0**.

	**Trigger**			**Precursor**		
	**2012**	**2013**	**2014**	**2012**	**2013**	**2014**
Beech	0.69	0.60	0.58	0.28	0.28	0.25
Pine	0.83	0.66	0.48	0.42	0.40	0.26
Oak	0.77	0.58	0.31	0.35	0.23	0.13

### 3.4. Coincidences of the timings of SRV events between the individual trees

**Figure 4:**
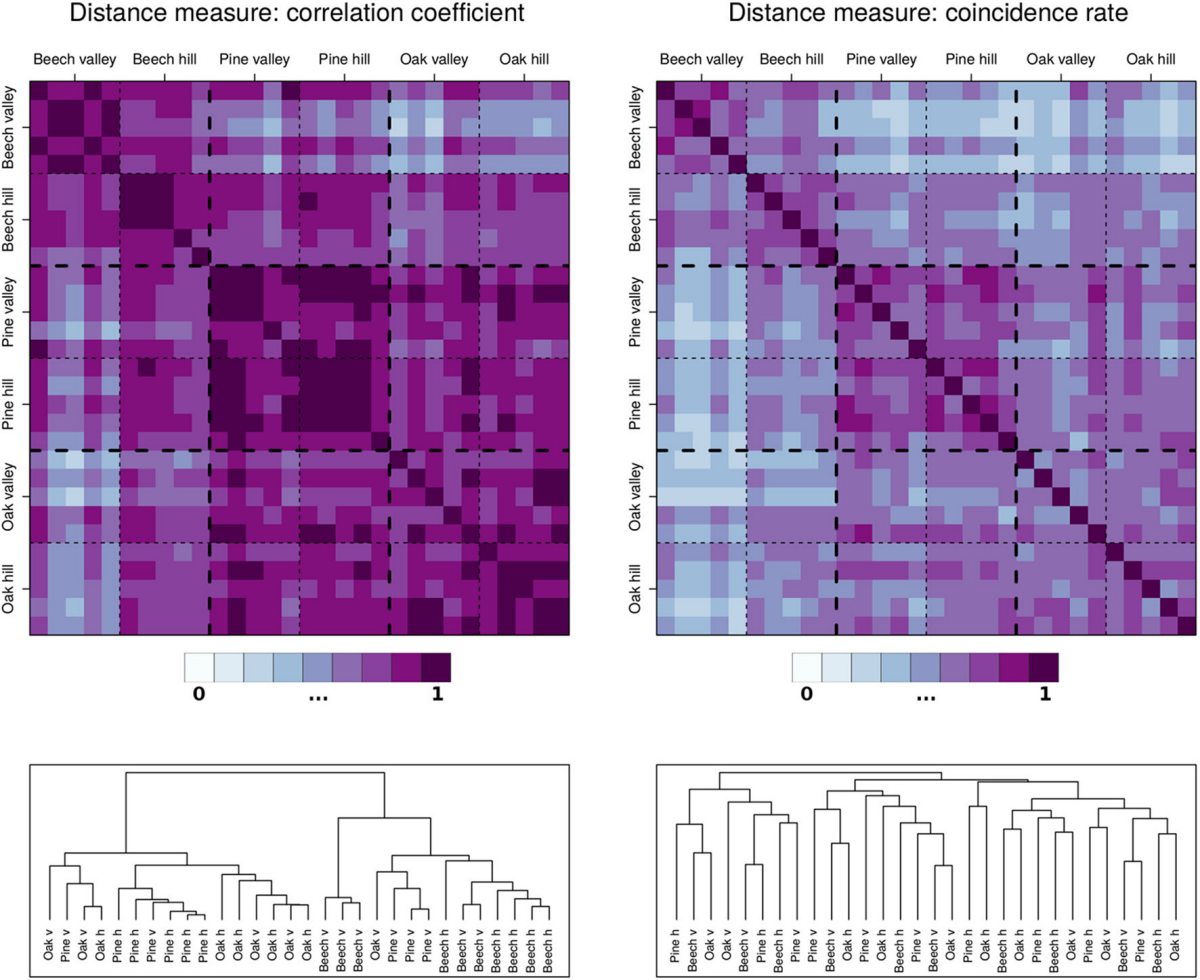
**Hierarchical cluster analysis for the 30 dendrometer time series of the growth periods of 2012, 2013, and 2014**. **Upper panels:** Correlation (daily stem increments) and coincidence (values above 90th percentiles) matrices between all pairs of trees used for the cluster analysis. **Lower panels:** Dendrograms of the two cluster analyses. The tree stands can be further subdivided into “hill” and “valley,” indicated by *h* and *v*.

When comparing the results of both bi- and multivariate ECA between the three tree species, the differences are relatively small. Altogether, oak seems to not favor wet conditions as strongly as beech and pine, but systematic inter-species differences appear to be absent. In turn, for the mean behavior (of daily as well as subdaily features), the growth characteristics are widely known to differ markedly between different tree species (Drew and Downes, 2009; Miralles-Crespo et al., 2010; Köecher et al., 2012; Butt et al., 2014). The question, whether there are differences or commonalities regarding the tree species' upper or lower parts of the distribution of SRVs has not been addressed to far. In order to investigate this issue for the three species of this study, we performed a hierarchical cluster analysis as described in Section 3. Figure 4 shows the results of this analysis and reveals that although the mutual correlations between the individual trees are quite high, the coincidence rates between days with positive SRV events are comparatively low. Based on correlation, we additionally find that the tree individuals are quite well clustered, while when using coincidence rates as similarity measure, the clusters following the individual species are completely lost. This means that the highest SRV variations of the individual trees vary strongly in their timing (at the daily scale), and that this timing is not generally differing by tree species. Hence, a clear systematic difference of the results between the different species (Section 3) cannot be found.

## 4. Discussion

### 4.1. Bivariate event coincidence analysis

The two broadleaved species show a positive response to temperature as well as LST while little positive response is found for pine. This positive growth response is likely due to the sufficient supply of water; possibly by reaching groundwater reservoirs. Similar relationships have been found earlier for the mean values (i.e., using correlation-based analyses) by van der Maaten et al. (2013) and others. In contrast to this, the fact that extraordinary negative *T*_*max*_ and/or *LST*_*max*_ events also coincide with positive SRV events (for all species) delivers important complementary information to the positive correlation between stem size variations and *T*_*max*_ found by van der Maaten et al. (2013) and Deslauriers et al. (2003). Due to the fundamentally different nature of these two statistical approaches, two time series can be positively correlated and simultaneously show significant coincidences between negative and positive events. This is not contradictory, but complementary information. The latter finding is further strengthened by the strong coincidence between positive *T*_*max*_ events and negative beech SRV events. Since both van der Maaten et al. (2013) and Deslauriers et al. (2003) performed their analysis for the entire (not subdivided) growing season, it is not possible to compare the seasonal timings of these contradicting behaviors.

Only few coincidences were found between SRV events and soil temperature extremes. It is likely that this observation is due to the location of the meteorological station 2 km from the dendrometer site. Due to the variability in soil type and ground cover throughout in the study area, actual soil temperatures beneath the sampled trees may systematically differ from the values measured at the station.

A positive instantaneous (lag zero) correlation between air humidity and SRV has been observed in previous studies (Downes et al., 1999; Deslauriers et al., 2003; Köecher et al., 2012; van der Maaten et al., 2013). In the present work, it was confirmed that this relationship is also evident for the upper (positive events) and lower (negative events) tails of the distribution of SRVs. As may be expected, a similar positive SRV coincidence is recorded with precipitation. However, the absence of a notable heavy precipitation impact during summer 2013 in all tree species is a result of a 60-days dry period except for one single day (35 mm). This suggests that one single heavy rainfall event is not sufficient to result in a significant coincidence rate even if it coincides with a marked positive or negative dendrometer anomaly (which was the case for almost all 30 trees).

The significant coincidences between low radiation values and positive SRV events can interpreted in a twofold way: One the one hand, low radiation decreases transpiration leading to water replenishment. On the other hand, low radiation days commonly correspond to cloudy and foggy conditions and are therefore often characterized by high relative humidity as well. A general negative dependency in terms of negative correlations between radiation and stem radius variability was reported earlier by Downes et al. (1999) and Köecher et al. (2012).

**Figure 5:**
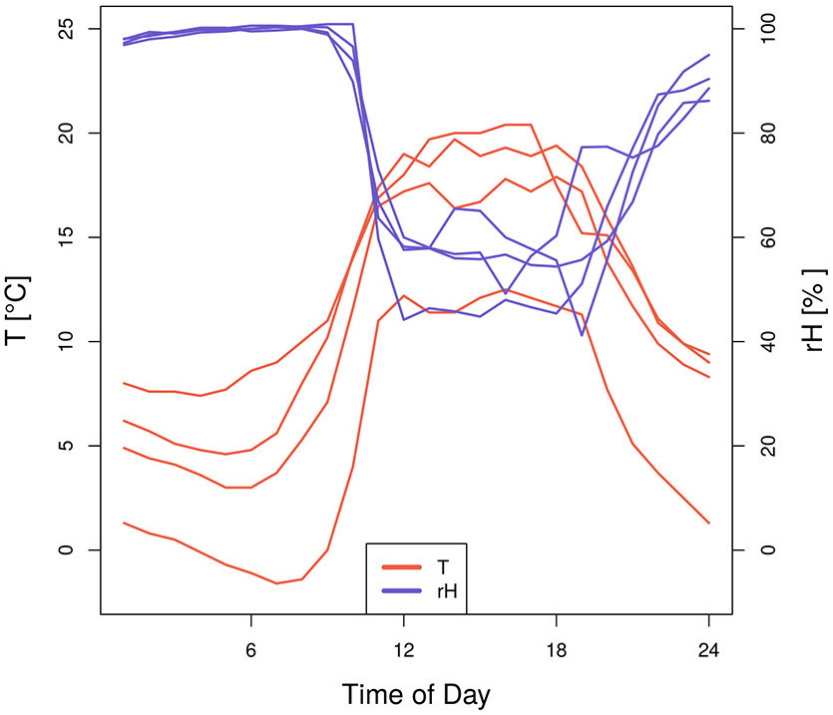
**Hourly values of temperature and relative air humidity of four selected days in spring and early summer 2014**. These days were all characterized by minimum temperature, maximum relative air humidity and negative SRV events.

Our analysis revealed some counter-intuitive significant coincidences between days with extraordinary high air humidity and negative SRV events in beech stems during 2014 (Figure 2). One possible cause for this may be the result of high air associated with low soil moisture conditions typical of foggy days during spring. Further support of this theory is provided by Figure S2, where in the upper right panel joint coincidences between low *T*_*min*_, high *rH*_*max*_ and negative beech SRV events are evident. In order to understand these joint coincidences in more detail, Figure 5 illustrates the temperature and air humidity development of 4 days in spring and early summer 2014, where the above mentioned coincidences appeared. Specifically, the figure shows very high air humidity values of up to 99% around 9–10 a.m. which abruptly decrease simultaneously with increasing temperature. This behavior is a common indication of foggy conditions during morning hours—caused by inverted atmospheric stratification—that are relieved by the rapidly increasing temperatures on a cloud-free day. The negative SRV events of these days are caused by the extraordinary high temperature and low humidity values of the mid-day and are not linked with the high air humidity values of the morning hours. Applying a classical linear correlation analysis between daily maximum relative humidity and daily SRVs, these days would produce strong residuals, clearly deviating from the well-known positive statistical interrelationship between these two variables. Therefore, these specific days provide a very good example of how to explain singular large residuals in classically assumed interrelationships between weather conditions and dendrometer variations as concluded from correlation analysis on a daily basis.

The results of bivariate (Figures 2, 3), as well as multivariate (Figures S1–S6) event coincidence analysis show that for a number of variables, coincidences with SRV events are not equally evident during all the 3 years of the investigation period. One example was mentioned in the previous paragraph. Another example is that positive *T*_*max*_ events only coincide with negative beech SRV events in 2012 and 2013, but not in 2014 (Figure 2, upper right panel). In this case, the reason is, that the positive *T*_*max*_ events in 2014 are, in absolute values, lower than the positive *T*_*max*_ events of the previous 2 years. Even though the events were defined by percentiles over the entire investigation period, a high number of positive *T*_*max*_ events in 2012 and 2013 led to this non-uniform distribution of events. In other words: in 2014, the highest *T*_*max*_ events were not warm enough to trigger negative SRV events. The same explanation is also valid for the absence of coincidences between positive SRV events (for all species) with *LST*_*min*_, *ST*_*min*_ and *ST*_*mean*_ in 2012. While in these cases the heterogeneous distribution of coincidences among the years can easily be explained, in other cases the reasons are less obvious. One possible factor leading to inter-year differences in the relation between weather conditions and SRVs could be the effect of the previous year's weather conditions on the current tree growth. Corresponding impacts on earlywood production (especially for oak, but also for the other two species) have been reported by a variety of studies (Lebourgeois, 2000; Rubino and McCarthy, 2000; Lebourgeois et al., 2004, 2005; Drobyshev et al., 2008; Michelot et al., 2012; Latte et al., 2015). Yet, all previous analyses that the authors are aware of, addressed integrated/cumulative stem size rates in terms of total earlywood production. So far, no statistical evidence or physiological explanation has been reported, why and how previous year's conditions should influence the day-to-day SRVs and their reaction to weather extremes.

### 4.2. Extraordinary positive vs. negative stem size variation events

The counter-intuitive negative beech SRV events found in Figure 2 and discussed in Section 4.1 could also be explained by a statistical artifact due to the co-occurrence between negative SRV events and positive SRV events of the previous day. Such a phenomenon of contradicting climatic signals in tree rings has also been reported for high resolution tree-ring isotope data (Schollän et al., 2013, 2014).

The various cycles of swelling and shrinkage shown by dendrometer data have been addressed in several recent studies (Downes et al., 1999; Köecher et al., 2012; van der Maaten et al., 2013; Vieira et al., 2013). From previous studies like Bouriaud et al. (2005), it is known that stems can shrink over several consecutive days, likely induced by shrinkage of the bark and relative sap-flow reduction of the stem some time after rain events. Our study refined these findings by pointing out that there are two kinds of strongly positive stem size variations: (1) some that are followed by negative SRV events and (2) some that are not followed by negative stem size changes during consecutive days. For future studies it will be important to investigate how to disentangle the four possible combinations of strong stem size changes defined in this study: There are strong positive SRV events that are followed (i.e., neutralized) by negative SRV events vs. positive SRV events that permanently increase the stem radius, as well as negative SRV events that simply originate from strong positive SRV events during the previous days vs. negative SRV events that have been forced by adverse weather conditions. A first attempt to disentangle these distinct phenomena has been recently published by Chan et al. (2016). The mentioned classical approach do define growth and shrinkage in dendrometer data (Deslauriers et al., 2003; Bouriaud et al., 2005; Köecher et al., 2012; Vieira et al., 2013) does not solve this problem, since (as for the procedures used by van der Maaten et al. (2013) and also in this study) both shrinkage and growth are solely defined based upon the preceding evolution and do not take into account the (short or long-term) following development of the stem radius.

### 4.3. Joint event coincidence analysis

The JECA revealed six main findings common to all three investigated tree species. (i) The combination of high minimum temperature with high relative humidity events coinciding with positive SRV events describes situations of warm nights followed by moist days. This feature was most clearly visible for pine which is to be explained by pine having the highest potential for water storage due to its larger amount of xylem Pfautsch et al. (2015). (ii) The positive impact of various temperature combinations on stem radius is a logical continuation of the results of bivariate ECA as discussed above. (iii) The combination between low radiation and low maximum temperatures describes very cloudy days. Such days are often also characterized by high air humidity, and this combination (high humidity and low radiation) is highlighted by finding (vi). (iv) Heavy precipitation as an additional event contributing to the aforementioned cloudy and moderately cool days also favors strongly positive SRVs. Such days are often also characterized by high night temperatures (high *T*_*min*_) corresponding to situation (v) highlighted in Section 3.2.

### 4.4. Differences in the timing of stem size variation events between the species

The observations found in Section 3.4 are in fact not trivial, since very different reactions of the analyzed species to environmental conditions have been well documented by, e.g., Gonzalez-Munoz et al. (2014), Lévesque et al. (2013), Garcia-Suarez et al. (2009), or Kwiaton and Wand (2015). The difference of our analysis to these studies is, that our dendrometer analysis specifically takes into account the timings of SRVs and meteorological extremes on a daily basis, whereas previous studies analyzed relationships between tree stem growth and weather conditions on a seasonal time scale. Therefore, the finding that no clear species-to-species differences are evident in this study does not contradict studies on seasonal scales. In turn, our results suggest, that the species-specific relations on weather conditions are more clearly expressed on longer rather than on shorter time scales. Nonetheless, our study indicates, that the different species do not differ markedly in their susceptibility to climate extremes on a daily scale. General statements or even suggestions to forest management concerning the species' eligibility in the context of ongoing and future climate change should not yet be drawn from this first case study.

## 5. Conclusions

We have used high-resolution dendrometer data to investigate tree species-specific responses to extraordinary meteorological conditions. For the first time joint event coincidence analysis as well as a hierarchical clustering analysis based on coincidence rates have been used. This new approach allowed a detailed analysis of the timing of observations falling in the upper and lower tails of the empirical distributions of daily SRVs. This opens new possibilities for interpreting tree-specific responses to meteorological extremes. Our method is able to provide relevant complementary information beyond what has been known from previous correlation-based analyses. Further potential applications of this method include the investigation of dendrochronological data or intra-annual density fluctuations (IADF).

For future investigations, it will be crucial to put additional efforts into disentangling tree stem radius growth from stem swelling, using novel data analysis approaches. Additionally, integrated studies including dendrometer and wood density measurements, as well as an up-scaling across a larger area will be necessary to draw reliable conclusions on tree or forest carbon storage dynamics in relation to meteorological extreme events.

## Author contributions

JS Study's Design, Data Analysis, Figures, Writing. TS Study's Design, Proofreading. IH Data Production and Preprocessing, Proofreading. EV Proofreading. SS Data Production and Preprocessing. GH Study's Design, Proofreading. RD Study's Design, Writing, Proofreading.

### Conflict of interest statement

The authors declare that the research was conducted in the absence of any commercial or financial relationships that could be construed as a potential conflict of interest.
