# Supplementary material for: Meteorological Drivers of Extremes in Daily Stem Radius Variations of Beech, Oak, and Pine in Northeastern Germany: An Event Coincidence Analysis
Source: Front Plant Sci. 2016 Jun 3;7:733. doi: 10.3389/fpls.2016.00733 (PMC4891350; doi:10.3389/fpls.2016.00733)
Supplement: Supplementary file 1 [file Presentation1.pdf]

Supplementary Material:
Meteorological Drivers of Extremes in Daily Stem Radius Variations of Beech, Oak and Pine in Northeastern Germany: An Event Coincidence Analysis

Jonatan F. Siegmund [1,2,*], Tanja Sanders [3], Ingo Heinrich [4], Ernst van der Maaten [5], Sonia Simard [4], Gerdhard Helle [4] and Reik V. Donner [1]
*Correspondence:
Jonatan F. Siegmund
siegmund@pik-potsdam.de

SUPPLEMENTARY FIGURES

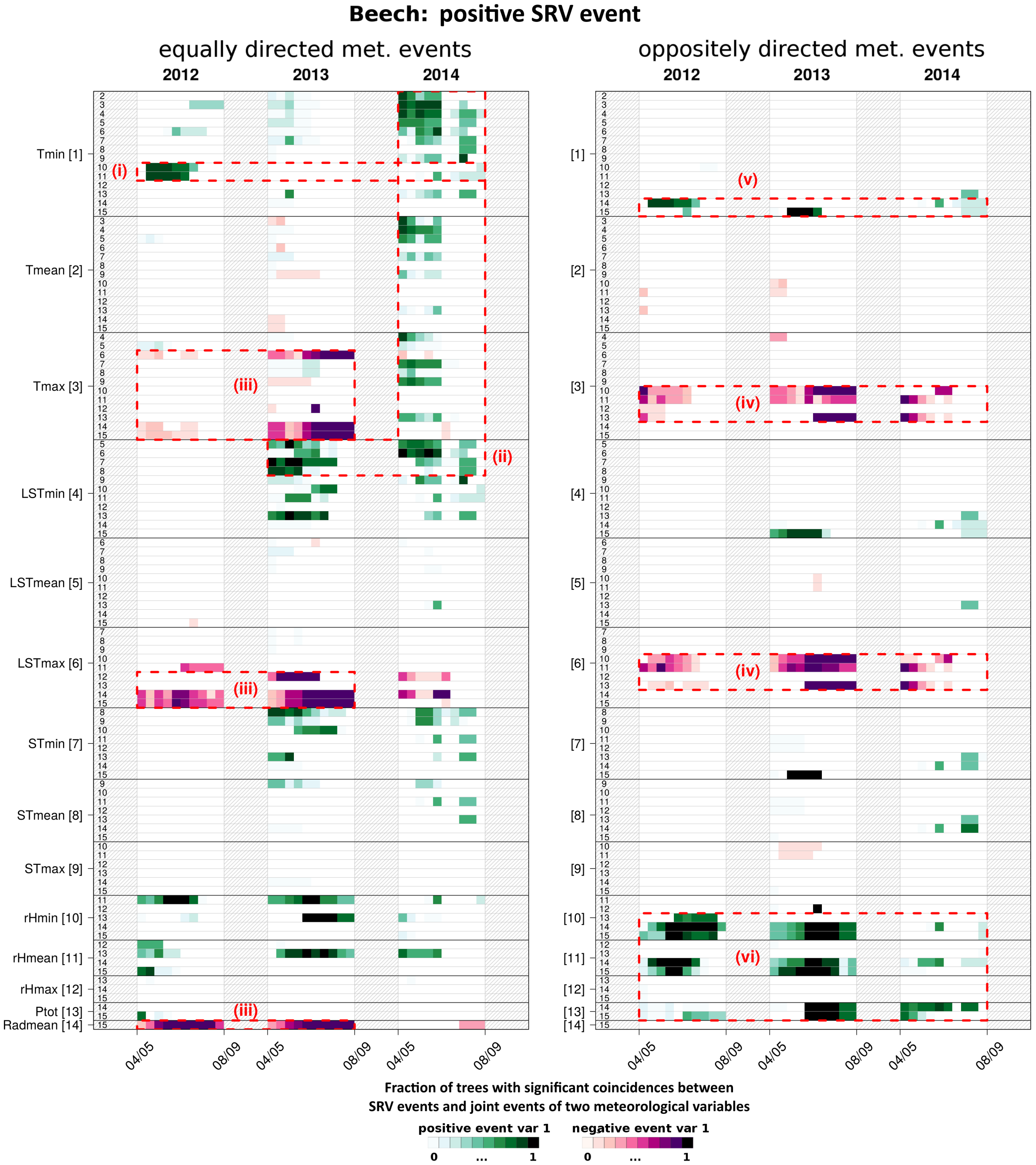

Figure S1. Coincidences between positive daily beech SRV events and joint positive/negative events of pairs of meteorological variables ($\Delta T = \tau = 0$). The left (right) panels show the results for equally (oppositely) directed meteorological events. Colors refer to the direction of the meteorological event in the first variable (indicated at the left) – green colors refer to positive and purple colors to negative events. The respective second variable is indicated by small numbers inside the individual panels (as explained at the left) and has either the same (left panels) or opposite (right panels) directionality of the extreme. Parts of the plots enclosed by dashed red lines highlight the six main findings (i)-(vi) discussed in the main text.

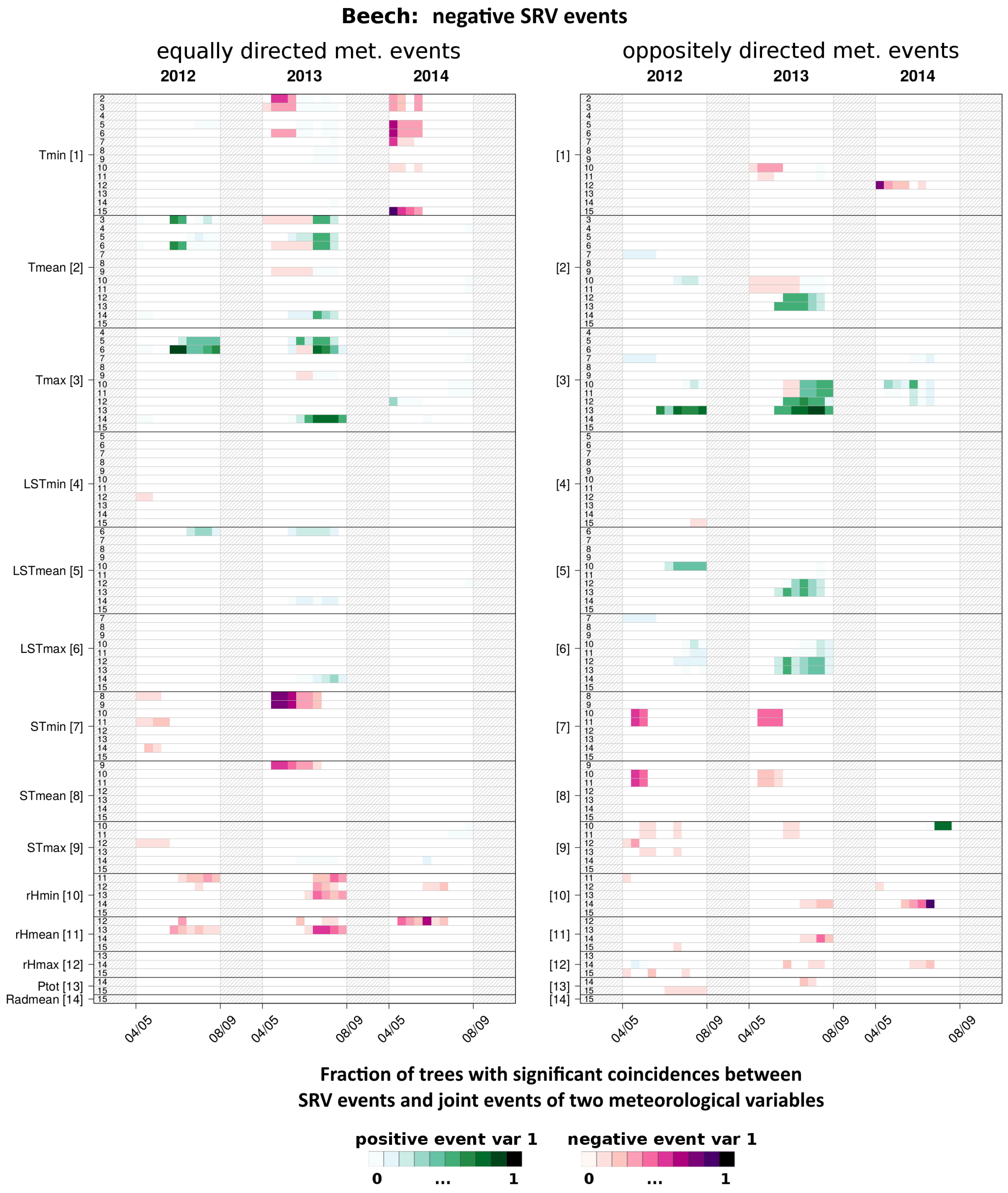

Figure S2. As in Fig. S1 for strongly negative beech SRVs.

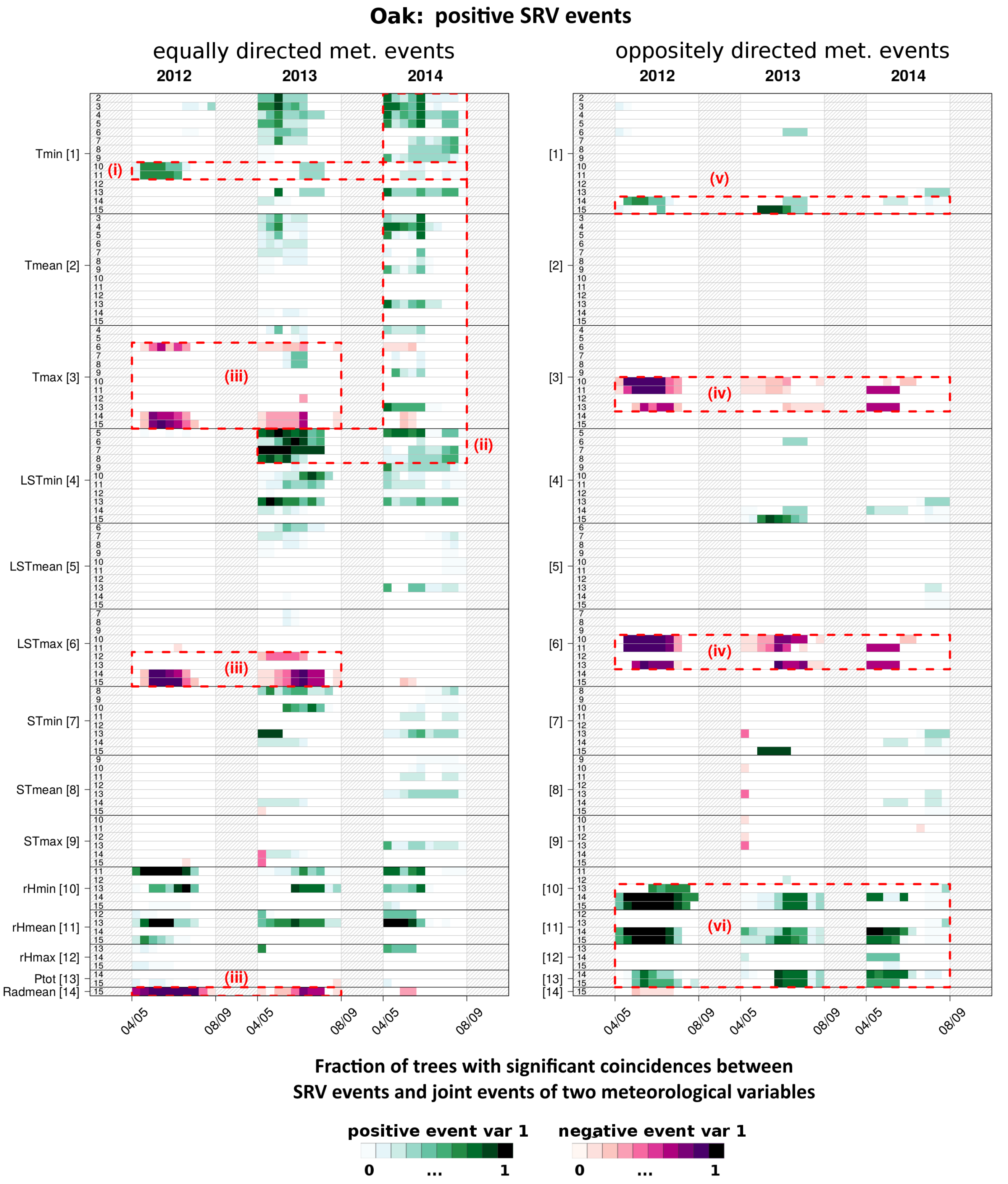

Figure S3. As in Fig. S1 for strongly positive oak SRVs.

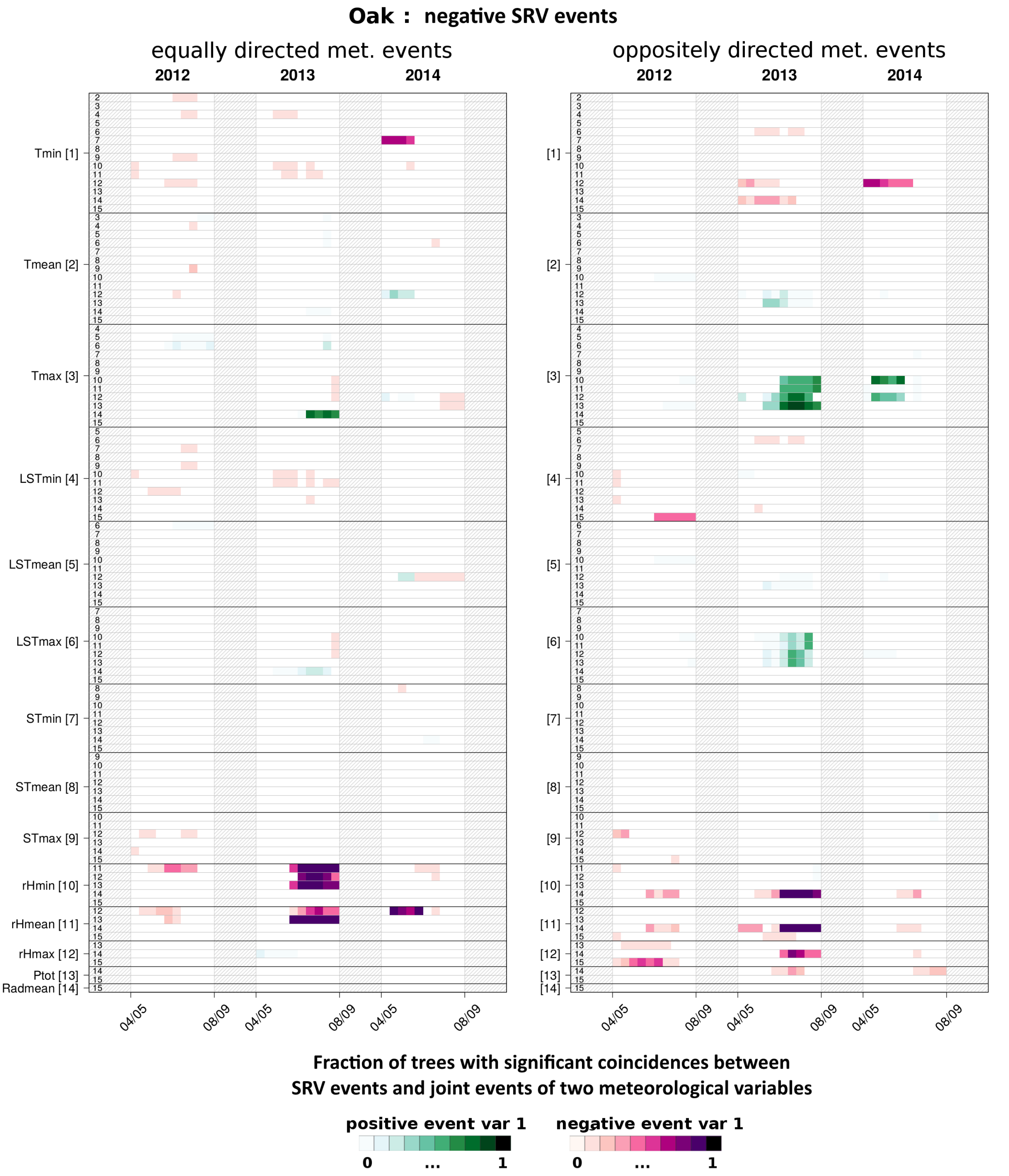

Figure S4. As in Fig. S1 for strongly negative oak SRVs.

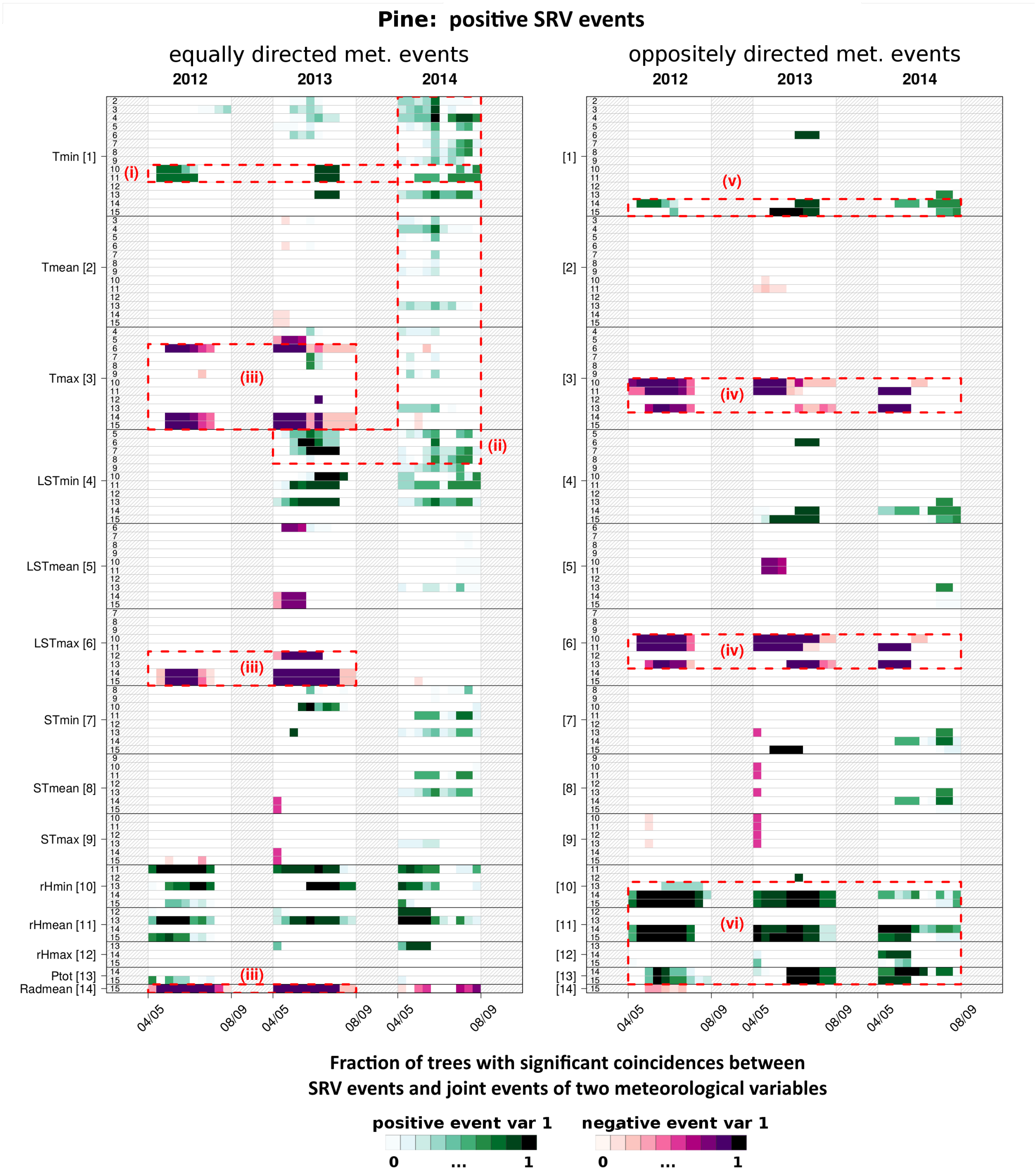

Figure S5. As in Fig. S1 for strongly positive pine SRVs.

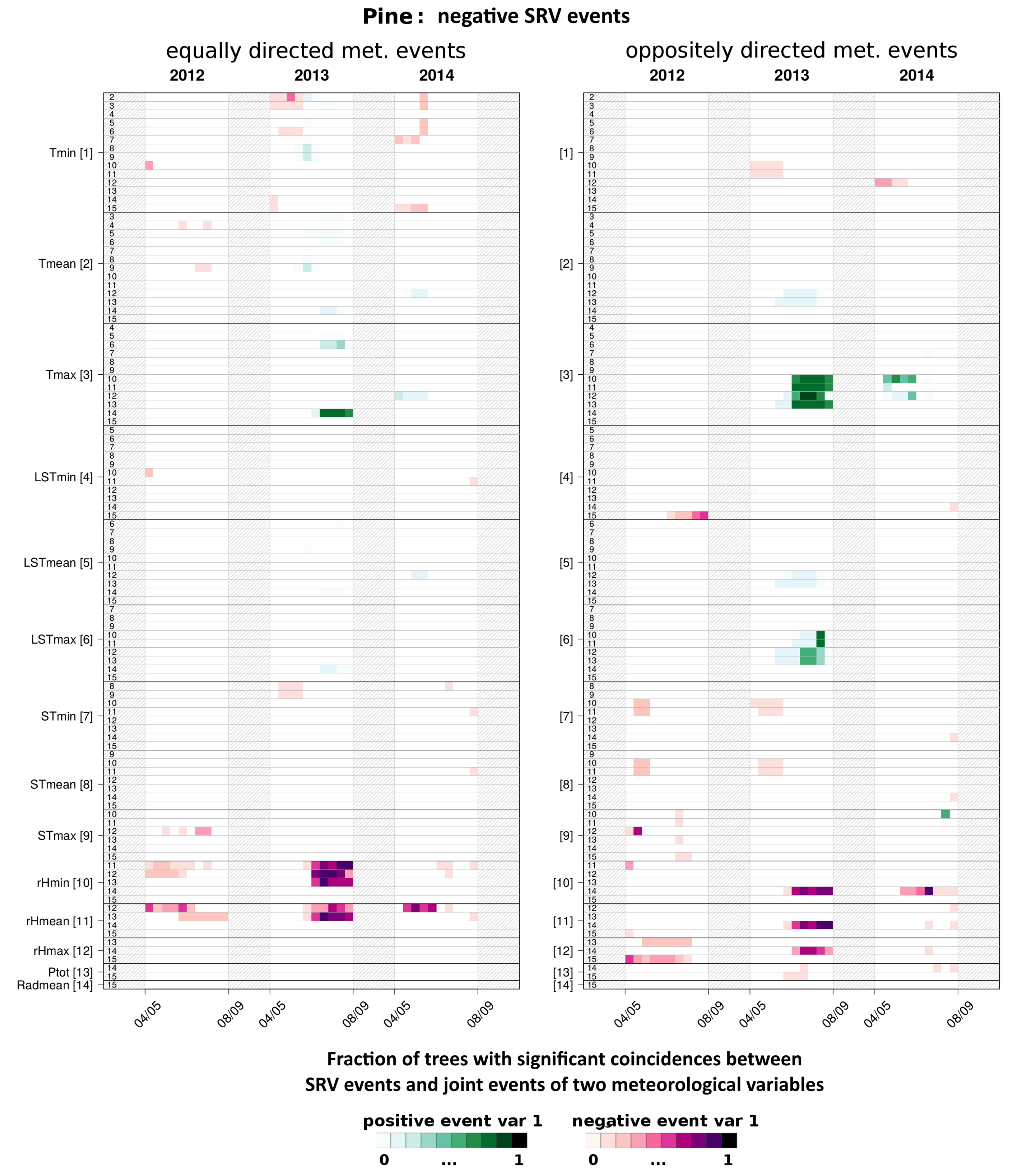

Figure S6. As in Fig. S1 for strongly negative pine SRVs.
